# P-1774. Order sets paired with facilitation training to effectively reduce postoperative surgical prophylaxis

**DOI:** 10.1093/ofid/ofae631.1937

**Published:** 2025-01-29

**Authors:** Virginia McKay, Collin McGovern, Jacqueline M Saito, Shawn Rangel, Kelly Bono, Jason G Newland, Sara Malone

**Affiliations:** Washington University in St. Louis, Saint Louis, MO; Washington University in St. Louis, Saint Louis, MO; Children's National Hospital, Washington, District of Columbia; Boston Children's Hospital/Harvard Medical School, Boston, Massachusetts; Washington University School of Medicine, Saint Louis, Missouri; Washington University in St. Louis School of Medicine, St. Louis, Missouri; Washington University School of Medicine, Saint Louis, Missouri

## Abstract

**Background:**

Inappropriate surgical antibiotic prophylaxis occurs in up to 40% of cases, primarily reflecting unnecessary postoperative doses ( >24hrs) in clean or clean-contaminated cases. It accounts for approximately 20% of all inappropriate use in children’s hospitals. We described order set outcomes from the Operatic Trial to compare two strategies, order set changes versus order set change with facilitation, to reducing excessive postoperative prophylaxis.

**Figure 1.**
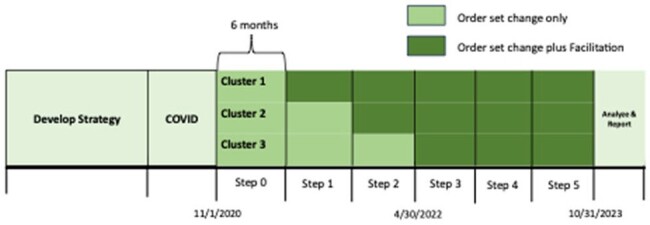
Stepped-wedge trial design comparing order set change only (control) versus order set change plus a facilitation workshop (intervention) attended by the ASP team at the beginning of each step. Nine children’s hospitals were randomized to 1 of 3 cluster. Each cluster included 3 hospitals and each step was 6 months in duration.

**Methods:**

The operatic trial was a stepped-wedge cluster randomized control trial with nine US children’s hospitals (11/1/2020-10/31/2023), with 6 steps lasting 6 months each (Figure 1). Antimicrobial stewardship teams were recruited and enrolled. After 6 months of all hospitals starting in control (step 0) of changing order sets, 3 hospital ASP teams in steps 1-3 participated in a virtual facilitation workshop (int). Facilitation is a strategy that supports people in health services organizations develop the means to change the structure and processes within settings, and clinicians can be educated on the skills and expertise to become an effective facilitator. Hospital ASP teams were surveyed every 2 months to evaluate order set changes.

**Figure:**
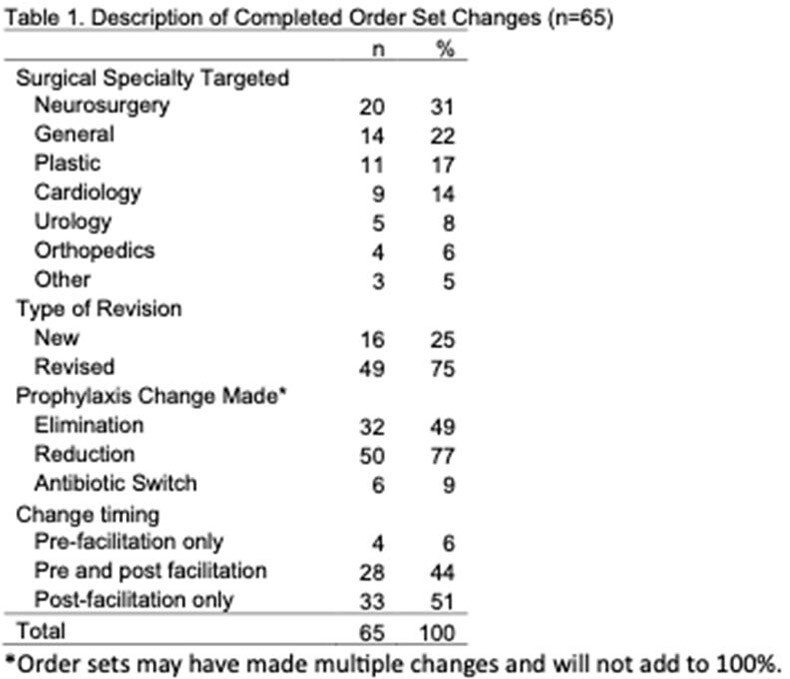

**Results:**

Approximately 75% of order set changes attempted were completed (65/85). Table 1 describes completed order set changes demonstrating a variety of specialties targeted and a focus on reducing prophylaxis duration. Importantly, only 4 were initiated and completed in the pre-intervention period, while the remaining were either initiated pre-intervention and completed post-intervention or initiated and completed post-intervention suggesting that facilitation training supported order set change completion. The median time to complete an order set change was 5 months. Individuals also reported other activities to support change, for example data dashboards and paper-based guidance.

**Conclusion:**

Changing order sets is an effective approach to reduce excess antibiotic use in children’s hospitals. Facilitation played a role in the successful completion of order sets, including potentially assisting teams to complete order set changes that were initiated prior to facilitation training.

**Disclosures:**

**Jason G. Newland, MD, MEd**, Moderna: Grant/Research Support|Pfizer: Grant/Research Support

